# Rehydration of the Tendon Fascicle Bundles Using Simulated Body Fluid Ensures Stable Mechanical Properties of the Samples

**DOI:** 10.3390/ma15093033

**Published:** 2022-04-21

**Authors:** Sylwia Dabrowska, Krzysztof Grabowski, Andrzej Mlyniec

**Affiliations:** Faculty of Mechanical Engineering and Robotics, AGH University of Science and Technology, 30-059 Krakow, Poland; sd.dabrowska@gmail.com (S.D.); kgrabow@agh.edu.pl (K.G.)

**Keywords:** tissue hydration, water content, mechanical testing, ex vivo testing

## Abstract

In this work, we investigate the influence of dehydration and subsequent rehydration of tendon fascicle bundles on their structural and mechanical properties by using distilled water, 0.9% NaCl, 10% NaCl, SBF, and double concentrated SBF (SBFx2). The properties of tendon fascicle bundles were investigated by means of uniaxial tests with relaxation periods and hysteresis for samples with various interfascicular matrix content, dissected from the anterior and posterior areas of bovine tendon. Uniaxial tests with relaxation periods and analysis of sample geometry and weight showed that dehydration alters the modulus of elasticity dependent on the interfascicular matrix content and influences the viscoelastic properties of tendon fascicle bundles. Tensile and relaxation tests revealed that changes resulting from excessive sample drying can be reversed by rehydration in an SBF bath solution for elastic strain range above the toe region. Rehydration in SBF solution led to minor differences in mechanical properties when compared to control samples. Moreover, anterior samples with greater interfascicular matrix content, despite their lower stiffness, are less sensitive to sample drying. The obtained results allow us to limit the discrepancies in the measurement of mechanical properties of wet biological samples and can be useful to researchers investigating soft tissue mechanics and the stability of transplant materials.

## 1. Introduction

Tendons are connective tissues which transmit a load from muscles to bone and are often overloaded, resulting in injury and tendon rupture. Analysis of the mechanisms of damage and disease processes requires both in vivo and ex vivo testing [1,2,3,4,5]. For reliable research, ex vivo samples should correspond to their in vivo counterparts. However, numerous parameters which influence the biological or mechanical outcomes make it difficult to derive any experimental rationale through ex vivo testing [6]. Moreover, biomaterials such as tendons undergo rapid dehydration throughout the ex vivo test procedure itself (preparation of samples, mounting, testing, etc.), thus radically altering the mechanical response of the tissue [7,8]. This manifests as unpredictable mechanical behavior of the tendon samples. In the literature, discrepancy of the tendon elastic modulus reaches 40-fold, which may be caused by inter-individual differences in addition to hydration of the measured samples [9].

The effect of tissue hydration on the measured parameters has been investigated in various studies [10,11,12,13,14,15,16,17,18]. Masic et al. [10] showed that water is an integral part of the collagen molecule, which directly influences mechanical properties of the tendon. The effect of long-term exposure of rat tail tendon to various concentrations of NaCl, sucrose, and polyethylene glycol was investigated in [11]. The authors concluded that buffer solutions such as NaCl and sucrose are inappropriate for long-term mechanical tests due to a change in tendon mechanical properties after incubation. Bloom et al. [12] investigated the influence of PBS, polyethylene glycol, and saline bathing solutions on tendon microstructure and mechanics and reported that PBS causes microscale damage which is visible as non-recoverable fibril sliding. Kemp et al. [13] concluded that dehydration creates artifacts in biological samples which require water. This must be taken into consideration when investigating collagen-based tissues, especially when characterizing disease-induced differences. A study by Shahmirzadi et al. [15] showed that dehydration of tissue alters its mechanical properties. Specifically, dehydrated tissue shows slower stress relaxation. The authors also demonstrated that changes in tissue mechanical properties are related to hydration of extracellular matrix elastin and collagen. Hoffman et al. [16] investigated the influence of dehydration on cross-sectional area (CSA) and viscoelastic soft tissue properties. The changes in CSA were reversible upon rehydration; however, reduction in the creep rate during dehydration was not immediately recovered after rehydration. Grant et al. [17] revealed that the elastic modulus of collagen fibrils can be altered depending on the pH and solution concentration. Various interaction forces such as hydration, solvation, and hydrogen bonding between tropocollagen monomers could influence the elastic response.

The hydration of samples during ex vivo tests can be maintained by using various techniques. Test samples can be sprayed with physiological saline solution (0.9% NaCl) [19,20,21,22,23]. Alternatively, samples could be stored in sealed containers with high humidity (e.g. saturated steam) or humidity-controlled chambers [15,24]. However, if these methods cannot be used, it is necessary to submerge or cover the sample with saline-soaked gauze between the different stages of ex vivo testing [25]. Although there have been studies investigating the influence of tissue hydration on its biomechanical properties, the drying process and subsequent rehydration of tendon tissue (and its influence on their mechanical properties) is still poorly understood. Moreover, due to different compositions of the various solutions used for keeping biological samples hydrated and various amounts of interfascicular matrix within the tendon [19], the biomechanics of these samples can be difficult to compare.

The goal of our study was to investigate the influence of dehydration and subsequent rehydration of tendon fascicle bundles cut from two distinct areas of the tendon with differing IFM content. Posterior and anterior tendon bundles were removed from two different tendon areas containing 51.9 and 66.3% water, respectively. The goal of our study was to determine if modification of soft tissue water content is needed for reliable biomechanical testing by proper preparation of soft tissue samples before testing.

Analysis of dehydration and rehydration of tendon fascicle bundles showed that changes in viscoelastic properties resulting from sample drying depend on the IFM content and can be reversed by submersion in SBF bath solutions. Rehydration in SBF solution for 15 min restored the initial stiffness of tendon samples which were previously dried for 1 h. In addition to obtaining a similar modulus of elasticity, the samples regained their viscoelastic properties.

## 2. Materials and Methods

The tendon samples were analyzed immediately after excision and 15, 30, 45, and 60 min after excision, which reflects the longest time needed for sample preparation. Tensile tests with relaxation were performed for 0, 15, and 30 min. This is because longer drying times without rehydration result in excessive stiffness and brittleness of the samples.

For rehydration tests, the samples were dried for 60 min immediately after excision and then submerged in distilled water, 0.9% NaCl, 10% NaCl, SBF, and SBFx2 solutions (containing double the concentration of ions). Changes in sample CSA and weight during rehydration were monitored. Additionally, groups of samples were tested after rehydration for 15, 30, and 45 min in various solutions. Uniaxial tensile tests with relaxation periods and unloading were performed to investigate the alterations in viscoelastic properties caused by dehydration and rehydration of fascicle bundle samples.

### 2.1. Tendon Samples

Bovine tendons were received from a local abattoir (Cracow, Poland), purified of excess membranes, fat, and muscle tissue and then immediately secured by using wet gauze and frozen at −80 °C. Tendons were obtained, purified, and frozen on the same day. In this study, a total of 30 tendons were used, which were dissected from the hind limbs of healthy animals of a similar age (<2 years). The tests were performed on bovine deep digital flexor tendons (DDFT). Prior to testing, tendons were slowly thawed in two stages: for 4 h at 4 °C and then for 2 h at room temperature (23 ± 1 °C). Bovine tendon fascicle bundle samples were manually cut with a scalpel into segments of 90 mm in length and 4–6 mm in thickness and width. In our previous work, we showed that bovine DDFT exhibit different mechanical properties and have varied water content depending on their region [19]; thus, we removed fascicle bundles from posterior and anterior sides, which have differing water content. Each test was performed for bovine tendon fascicle bundles cut from the posterior side (*n* = 10) and anterior side of tendons (*n* = 10). The CSA of each fascicle bundle sample was measured by using a non-contact 3D blue laser scanning system, which we developed in our laboratory [26].

### 2.2. Determination of the Change in Dimensions and Weight of Samples during Drying

Immediately after excision, the free ends of each tendon sample were mounted in specially designed grips and installed onto the 3D scanner system [26]. Scans were performed immediately after the excision and after 15, 30, 45, and 60 min. Additionally, the sample was weighed by using an analytical laboratory scale (Radwag, AS 220.R2, 227 readability 0.1 mg, Radom, Poland). We analyzed percentage changes in the mean CSA of the samples and changes in sample weight in order to evaluate dynamics of the drying process.

### 2.3. Determination of the Change in Dimensions and Weight of Samples during Drying

Immediately after excision, the free ends of each tendon sample were mounted in specially designed grips and installed onto the 3D scanner system [26]. Scans were performed immediately after the excision and after 15, 30, 45, and 60 min post-excision. Additionally, the sample was weighed by using an analytical laboratory scale (Radwag, AS 220.R2, 227 readability 0.1 mg, Radom, Poland). We analyzed percentage changes in the mean CSA of the samples and changes in sample weight in order to evaluate dynamics of the drying process.

### 2.4. Sample Group for Mechanical Testing

In mechanical tests, the control group consisted of samples which were scanned with a 3D laser immediately after cutting and subjected to stretching tests with relaxation periods. In the first stage of the investigation, mechanical tests were carried out on samples which were left in laboratory conditions at room temperature (23 ± 1 °C) for 15 or 30 min after cutting. In the second stage of the investigation, mechanical tests were performed on samples which were left at room temperature for 60 min after cutting and then rehydrated in an appropriate solution. The following solutions were used for rehydration: distilled water, 0.9% NaCl, 10% NaCl, SBF, and a concentrated SBFx2 solution. The SBF was prepared according to Kokubo [27]. Each sample was incubated separately in a closed test tube.

### 2.5. Mechanical Test-Tensile Tests with Relaxation Periods

Directly before testing, each fascicle bundle sample was mounted between grips with similar force and then scanned by using a 3D scanning system to determine CSA [26]. The scanning process took approximately 4 min. Grip-to-grip length was approximately 55 mm. Immediately after scanning, the samples were mounted on the testing machine (INSTRON 8872), which is a uniaxial servohydraulic strength machine (Instron, Norwood, Massachusetts, USA) equipped with a load cell having a maximum capacity of 250 N [19]. The mechanical test was initiated by preloading the samples with a constant force of 2 N, followed by a preconditioning process with 10 sine cycles of 1 Hz frequency in the strain range between 0 to 2%. The samples were then stretched with 1% s^−1^ strain rate to 2, 4, and 6% of strain. Between each tensile step, samples were subjected to 30 s of stress relaxation. This allowed us to evaluate the nonlinear viscoelastic properties of the tendon samples. Changes in elastic modulus, relaxation rate, and viscoelastic properties were analyzed.

The elastic modulus was estimated as the tangent to the stress-strain curves, at 2%, 4%, and 6% of strain [19]. The stress value of the stress-strain curves was determined by using the CSA of each sample and loading data from a tensile machine. Analysis of sample viscoelastic properties was carried out based on the level of relaxation from stress-time curves after obtaining 2%, 4%, and 6% of strain. The rate of relaxation was calculated as the normalized relative percentage value of stress drop obtained during 30 s of relaxation. The viscoelastic nature of tendons was also estimated in terms of energy dissipation, which was calculated as the area of a hysteresis loop in a stress-strain loading-unloading curve. The normalized percentage value of energy dissipation was calculated as the ratio of the inner area of the hysteresis loop relative to area under the load curve.

### 2.6. Statistical Analysis

Results of mechanical analyses were expressed as mean ± standard deviation. The compliance with the normal distribution of all variables in the study was verified by using the Shapiro–Wilk test of normality. Statistical analyses were performed by using OriginPro (v2019, OriginLab Corporation, Northampton, MA, USA) software according to one-way analysis of variance (ANOVA) with Tukey’s test. The significance level was set to 0.05.

## 3. Results and Discussion

The Shapiro–Wilk distribution normality test was performed for all variables present in the study, i.e., cross-sectional area [mm^2^], weight [mm], modulus of elasticity [MPa], normalized value of hysteresis [%], and normalized stress drop [%]. Results from the Shapiro–Wilk test showed that for all parameters, the distribution in the entire sample is consistent with a normal distribution.

### 3.1. Influence of Dehydration Time on Biomechanical Properties of Fascicle Bundles

**Figure 1:**
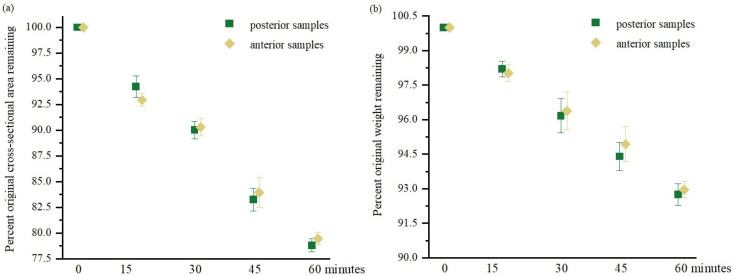
Change in cross-sectional area (**a**) and change in weight of fascicle bundles (**b**) during time (mean ± standard deviation). Values are given in percentage relative to the initial area measurement and weight immediately after excision (0 min); *n* = 10.

The influence of tendon fascicle bundle dehydration was evaluated after 15, 30, and 45 min of drying. The gradual decrease in CSA and sample weight are shown in Figure 1. After 15 min of drying, we observed a decrease in CSA and weight of the anterior samples equal to 5% and posterior samples equal to 2%. The greater reduction in CSA and weight of anterior samples, although not statistically significant, can be explained by its greater initial water content (66.3%), which is 14.4% greater than the water content of posterior samples [19]. Further drying of the samples for 30, 45, and 60 min resulted in 4%, 6%, and 8% weight reduction and 10%, 17%, and 21% CSA reduction, respectively (Figure 1). We did not observe a statistically significant reduction in CSA and weight between anterior and posterior samples. However, CSA and weight reductions were statistically significant (*p* < 0.001) between all drying times. This extended drying led to a greater decrease in CSA and weight of posterior samples, which correlated with results of our mechanical tests.

**Figure 2:**
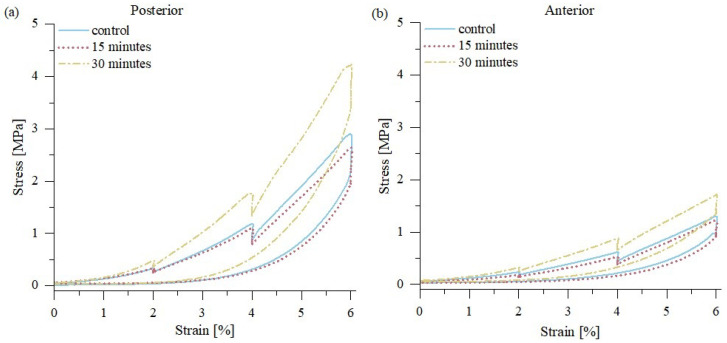
Mean stress-strain curves and standard deviation for the tendon samples taken from (**a**) posterior side and (**b**) anterior side of the tendon; *n* = 10.

Mechanical tests showed that sample drying causes significant changes in the mechanical response of the tissue on external loading (Figure 2). Uniaxial tests of tendon fascicles with relaxation periods showed that 15 min of drying led to a slight decrease in the elastic modulus, as well as the maximum stress for both posterior and anterior samples. This was the same for all three strain levels of 2, 4, and 6%.

**Figure 3:**
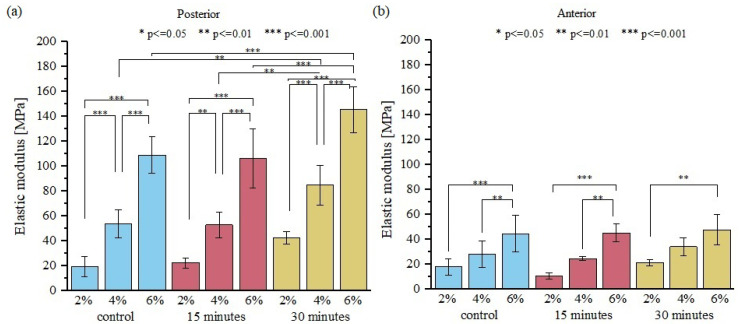
A comparison of elastic modulus obtained for (**a**) posterior side and (**b**) anterior side of the tendon for three different strain range/strain values for control samples and for samples dried for different times; *n* = 10.

**Figure 4:**
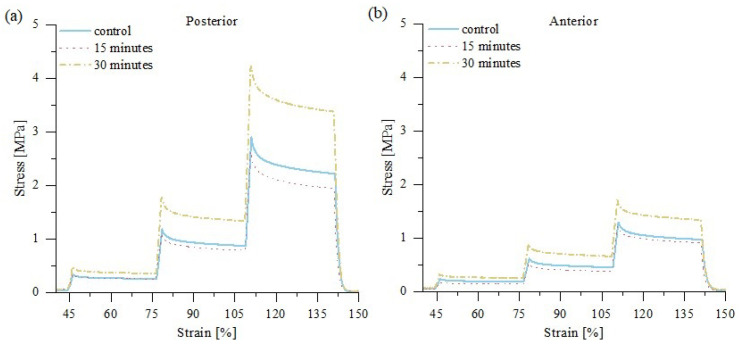
Mean stress-time relaxation curves after 0, 15, and 30 of drying for (**a**) posterior tendon fascicle bundles; (**b**) anterior tendon fascicle bundles. Visible increase in stiffness for both posterior and anterior samples; *n* = 10.

Further drying of the samples caused a significant increase in the elastic modulus (Figure 3), as well as the maximum stress, (Figure 4) which was approximately 50% greater than in the control group. The stress-time curves from uniaxial tension of posterior and anterior fascicle bundles, as shown in Figure 4, suggest that all samples exhibit the same qualitative behavior. Our presented results are consistent with previous studies, which showed that the change from moist soft tissue conditions to dehydrated air conditions increases the modulus of elasticity [7,17,28,29]. Moreover, mechanical changes during tissue dehydration may also be caused by the shrinkage of fibers during drying [10]. Comparing the strength properties of posterior and anterior sample areas of the tendon showed a significant (*p* < 0.001) increase in the elastic modulus and maximum stress only for posterior samples, which are characterized by lower water content (and consequently IFM content) and greater stiffness and damping properties than anterior samples [19]. This indicates that sample drying exerts a greater effect on the mechanical properties of tendon fascicles when compared to the IFM, which binds the fascicles together. Thus, tendon fascicle bundles excised from the anterior side have more stable elastic properties during sample drying.

**Figure 5:**
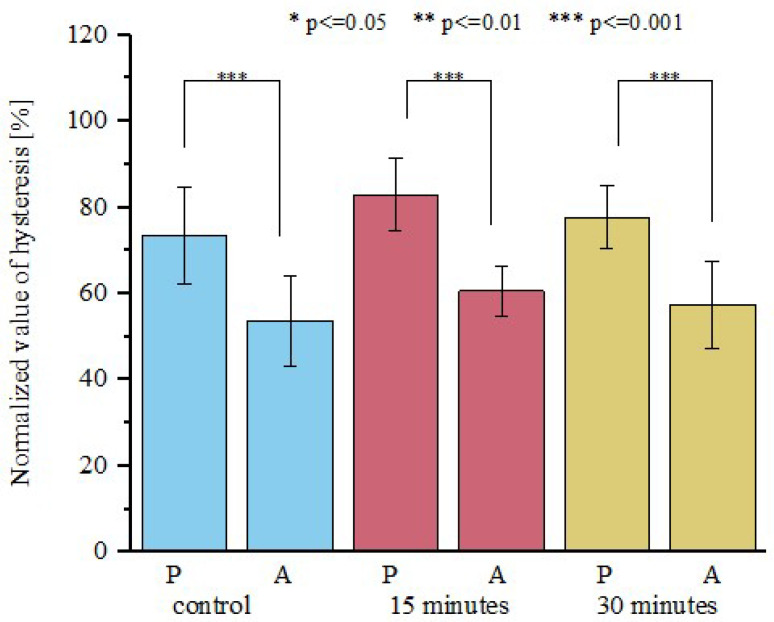
Normalized values of hysteresis for posterior and anterior tendon fascicle bundles. Values are shown for control samples, as well as for samples which were open-air dried for 15 and 30 min. Drying does not influence energy dissipation of the material; *n* = 10.

Analysis of the hysteresis for fresh and open-air dried fascicle bundles showed an increase in energy dissipation for anterior and posterior areas (Figure 5). However, the ANOVA test showed no statistically significant differences in energy dissipation between fresh and open-air dried samples.

**Figure 6:**
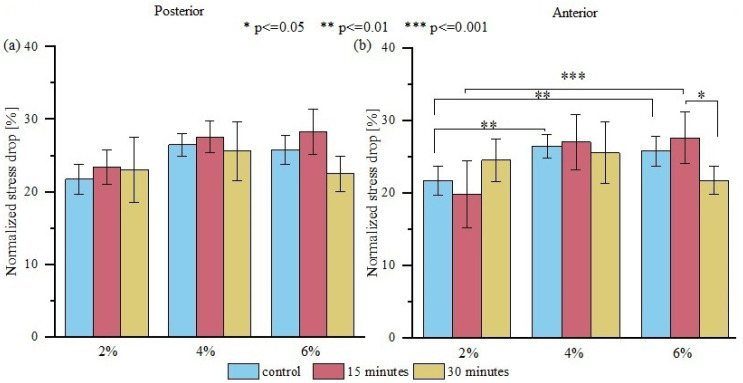
A comparison of normalized stress drop obtained for (**a**) posterior tendon fascicle bundles and (**b**) anterior tendon fascicle bundles for 2, 4, and 6% of initial strain. Values are shown for the control group and for samples dried for 15 and 30 min in open air. The viscoelastic properties of anterior samples were altered after drying; *n* = 10.

To evaluate the viscoelastic properties of the samples, each stress-time curve was normalized. We observed that the mean value of normalized relative stress drop for all analyzed groups from the posterior side exhibited similar normalized stress drop at 2, 4, and 6% (Figure 6a). The analysis of variance showed statistically significant differences between values of normalized stress drop at 6% of strain (Figure 6b) between anterior samples dried for 15 and 30 min (*p* < 0.05). Interestingly, anterior samples, which showed better elastic property stability during drying, are less stable in terms of viscoelastic properties. The greater change in the viscoelastic properties of anterior samples results from their greater water content and greater amount of IFM, which control the sample viscoelasticity through the flow of fluids within the material.

Our results show that dehydration of stiff samples with low water content (from the posterior side of the tendon) causes an increase in the modulus of elasticity but does not significantly change the viscoelastic properties. On the other hand, samples with high water content (cut from the anterior side of the tendon) have elastic properties less prone to drying, although the viscoelastic response depends on sample drying.

### 3.2. Influence of Fascicle Bundle Rehydration on Its Mechanical Properties

**Figure 7:**
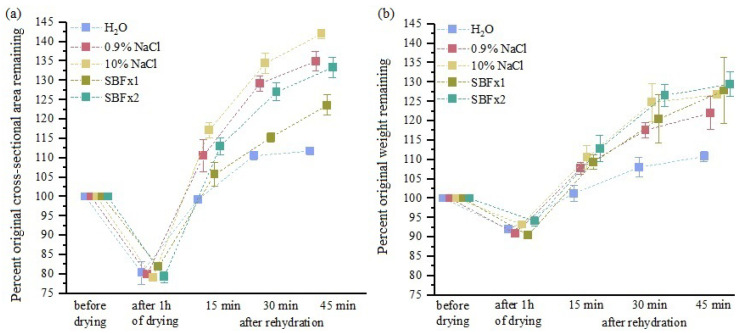
Changes in (**a**) cross-sectional area and (**b**) the weight of fascicle bundles during 60 min of drying followed by rehydration (mean ± standard deviation). Values are given in percentage relative to the initial area measurement and weight immediately after excision. (before drying); *n* = 10.

Changes in CSA values and sample weights during drying and rehydration in distilled water, 0.9% NaCl, 10% NaCl, SBF, and SBFx2 solutions are summarized in Figure 7. Sample drying for 60 min results in a 20% reduction in CSA and approximately 8% in sample weight, which are reversed after the first 15 min of rehydration in bath solution. The values of CSA and sample weight closest to their initial value are for samples rehydrated in distilled water and SBF bath solution. On the other hand, the greatest increase in CSA and sample weight was observed after 15 min of rehydration in concentrated SBFx2 and 10% NaCl hypertonic solutions. Extending the rehydration time results in a further increase in the CSA and weight of fascicle bundles.

**Figure 8:**
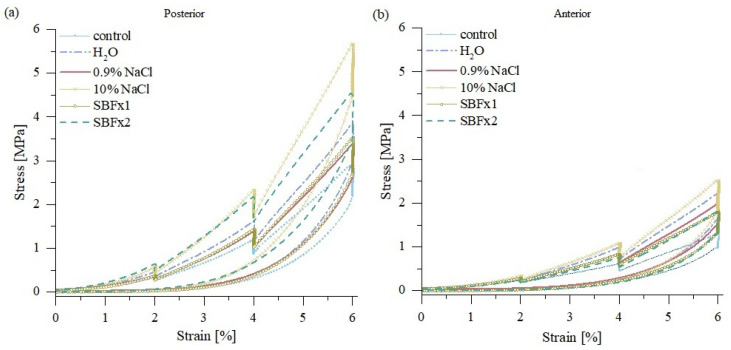
Mean stress-strain curves and standard deviation for bovine fascicle bundle samples taken from (**a**) posterior side and (**b**) anterior side of the tendon for control samples and after 15 min of rehydration in distilled water, 0.9%NaCl, 10% NaCl, SBF and SBFx2; *n* = 10.

**Figure 9:**
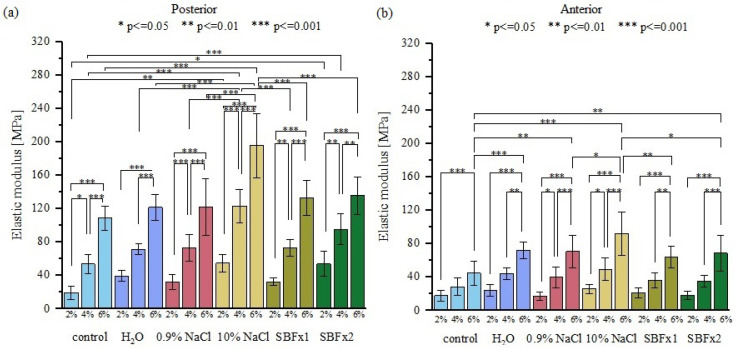
A comparison of the elastic modulus obtained from (**a**) posterior side and (**b**) anterior side of the tendon for three different values (2, 4, and 6%) of strain. Elastic modulus is given for control samples and for samples rehydrated for 15 min in distilled water, 0.9% NaCl, 10% NaCl, SBF, and SBFx2; *p* < 0.05; *n* = 10.

Uniaxial tensile tests (Figure 8) revealed that 15 min of rehydration in distilled water, 0.9% NaCl, and SBF do not result in significant changes in the elastic/tangent modulus for posterior samples (Figure 9a). On the other hand, the elastic modulus of anterior samples changed significantly for all bath solutions at 6% of strain, although SBF bath solutions induced the smallest changes (Figure 9b).

**Figure 10:**
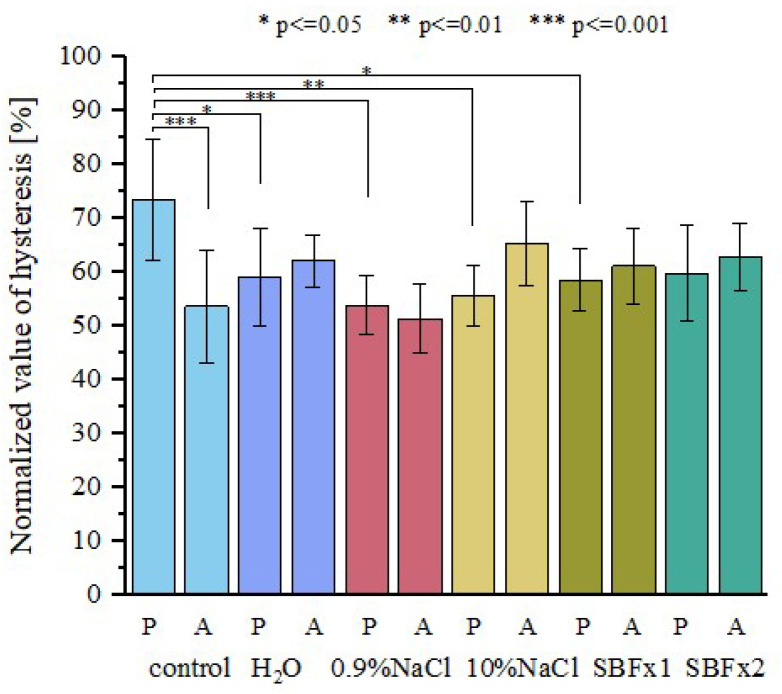
Normalized values of energy dissipation for anterior and posterior side of the tendon; *p* < 0.05; *n* = 10.

Analysis of the hysteresis for fresh and rehydrated fascicle bundles showed that the decrease in energy dissipation is observed only for the posterior area (Figure 10). The ANOVA test revealed a statistically significant decrease in values of hysteresis for samples rehydrated in water (*p* < 0.05), 0.9% NaCl (*p* < 0.001), 10% NaCl (*p* < 0.01), and SBF (*p* < 0.05). The lack of significant effects from hydration in concentrated SBFx2 solution on energy dissipation results only from significant scattering of the measured values.

The ANOVA test did not show any statistically significant differences between rehydrated anterior samples. Moreover, differences in energy dissipation between anterior and posterior samples became statistically non-significant after rehydration.

**Figure 11:**
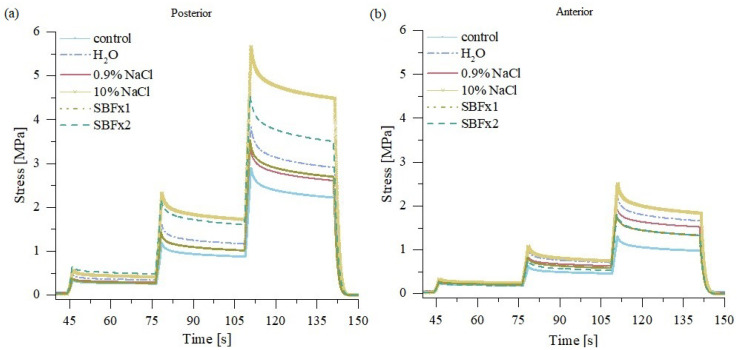
Mean stress relaxation curves for samples from the (**a**) posterior side and (**b**) anterior side of the tendon; for control samples and samples rehydrated in different solutions; *n* = 10.

**Figure 12:**
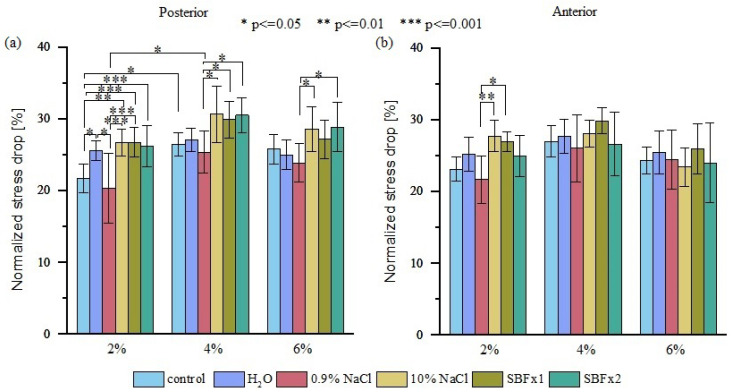
A comparison of normalized stress drop obtained from the (**a**) posterior side and (**b**) anterior side of the tendon for three different strain values. The normalized stress drop given for the control group and for rehydrated samples after 15 min of rehydration in distilled water, 0.9% NaCl, 10% NaCl, SBFx1, and SBFx2, *p* < 0.05.

Sample relaxation tests after rehydration (Figure 11) showed that normalized stress drop relative to the control group was statistically significant only for posterior samples at 2% of strain (Figure 12) when rehydrated in distilled water (*p* < 0.05), 10% NaCl (*p* < 0.01), SBF (*p* < 0.001), and SBFx2 (*p* < 0.001).

If rehydration alters energy dissipation and relaxation behavior only for posterior samples (which have less IFM), we can conclude that the bath solution affects only areas of high shear stress [30] generated between the fibrils during loading.

High stress states cause significant deformation of the sample, which may accelerate chemical processes [31], but above all, change the resistance of liquid flow inside porous materials. For anterior IFM-rich samples, uniaxial loading generates only small strains within the matrix, which does not alter fluid flow within the IFM, and thus, the viscoelastic response of the tissue remains unchanged. In posterior samples, which have less IFM than anterior samples, viscoplasticity of the matrix has a greater role, which does not depend on rehydration in a bath solution. Our studies provide experimental evidence that hydration level influences the biomechanics of samples differently, depending on the matrix content. Moreover, the mechanical response and structure of the tissue depends on the bath solution, which can influence tissue damping properties without altering its static elastic properties.

Our observations are in agreement with results published in [32], where the authors reported increased initial stress as well as slower stress relaxation in dehydrated samples. We observed this in posterior samples up to 15 min of drying.

Rehydration of samples in different aqueous solutions can lead to different intermolecular interactions through diffusion of solutes from the solution into the tissue [11,17]. Between neighboring tropocollagens, there may be both attractive and repulsive forces, which may cause changes in the mechanical properties [33,34] by increasing the hydraulic resistance of body fluid flow within the matrix.

Rehydration of samples in bath solutions with high ionic strength, such as 10% NaCl and SBFx2, causes a significant increase in the elasticity modulus and affects viscoelastic properties, as well as sample weight, especially for rehydration times longer than 15 min. Therefore, the key factor is not only the bath solution used but also the correct time selected appropriate to the size of the sample. Considering a 15-min rehydration time as sufficient to obtain the original weight of the sample, it can be concluded that the use of SBFx1 provides samples with properties closest to their initial values.

Rehydration tests showed that tendon fascicle drying, which occurs during sample preparation, is a reversible process and can be accomplished by immersing the sample in a bath solution. For the rehydration of soft tissue samples, saline and sucrose solutions, PBS or polyethylene glycol, are most commonly used . However, the use of these solutions clearly altered the biomechanical properties of tissue samples [11,12]. Optimization of the rehydration process requires taking into consideration not only the type of bath solution used and their ion concentration but also volume of the solution and sample weight and structure. Content of water and IFM within the tendon fascicle sample depends on the side of the tendon from which the sample was excised. This affects its behavior during both drying and rehydration. Therefore, to optimize the rehydration process, it is necessary to fully understand the dynamics of the rehydration process and to develop predictive models for arbitrary CSA and sample weight and structure. Development of predictive models can facilitate the selection of the proper concentration of bath solution and dehydration time, and can be implemented by using molecular dynamics (MD) [35], coarse-grained or reactive MD [31,36], or a structural-based continuum mechanics approach [37,38,39]. Development of this advanced predictive model requires not only a thorough knowledge of the tissue molecular structure but also the dynamics of body fluid flow through the IFM.

## 4. Conclusions

In the presented study, we investigated the differences in mechanical properties, CSA, and weight of tendon fascicle bundles affected first by dehydration, then followed by rehydration in 0.9% NaCl, 10% NaCl, SBFx1, and SBFx2 solutions. Our study revealed some noteworthy observations.

- Drying of tendon fascicle bundles in laboratory conditions caused a decrease in sample weight and CSA. The decrease in sample weight caused an initial decrease in sample stiffness (after 15 min) followed by a significant increase in stiffness after 30 min. This phenomenon is observed for both anterior and posterior sides of the tendon; however, a statistically significant difference was noted only for the posterior side, which has less IFM content and exhibits greater elastic modulus and damping, as observed in MRI and dynamic mechanical analysis [19].
- Relaxation tests for 2, 4, and 6% of strain only showed a tendency to increased normalized stress drop after 15 min of drying, followed by decreased normalized stress drop after 30 min. However, these were not statistically significant and should be understood as sample drying having only a limited influence on viscoplasticity of the IFM.
- Biomechanical changes induced by tendon fascicle bundle drying for 60 min can be reversed by 15 min of rehydration in SBF solution, which does not cause statistically significant changes for the selected range of interest.
- Tendon fascicle bundles dissected from the anterior side of the tendon, despite their greater IFM content and lower stiffness, are less prone to changes in tissue stiffness due to dehydration and rehydration.
- Rehydration bath solutions affect the mechanical properties of the tendon areas where one can observe high strain fields during loading. A greater IFM-to-fascicle ratio, visible on the anterior side of the tendon, minimizes the effect of bath solutions on the biomechanics of tendons.

Analysis of these results lead to another research question: How can we optimize the rehydration process to achieve repeatable measurement results, regardless of sample size and structure? Answering this question would allow us to ensure constant hydration of the tissue, which will allow us to limit the discrepancies in measurements of biological sample mechanical properties. Further research is necessary to develop predictive models which would allow optimization of the rehydration process. This requires a consideration of rehydration time and matching the bath solution volume to sample structure, size, and weight. Such a model will be useful for biomechanical engineers investigating soft tissue mechanics and for researchers studying the stability of transplant materials. Our data underline the need for adjusting the water content of soft tissue for biomechanical tests to optimize their importance.

## Data Availability

Data is available upon request from the corresponding author.
